# Preoperative use of furosemide may increase the incidence of acute kidney injury after coronary artery bypass grafting: a propensity score-matched study

**DOI:** 10.1007/s11748-021-01599-0

**Published:** 2021-02-06

**Authors:** Hui Zheng, Le Liu, Guoliang Fan, Zhigang Liu, Zhengqing Wang, Baocheng Chang

**Affiliations:** 1 NHC Key Laboratory of Hormones and Development, Tianjin Key Laboratory of Metabolic Diseases, ChuHsien−I Memorial Hospital and Tianjin Institute of Endocrinology‚ Tianjin Medical University, No. 6 Huanrui North Road, Ruijing Street, Beichen District, Tianjin, China; 2 Department of endocrinology, TEDA International Cardiovascular Disease Hospital, No. 61 Third Avenue, Tianjin Economic and Technological Development Zone, Tianjin, China; 3 Department of Geriatrics, The Second Hospital of Tianjin Medical University, Tianjin, China; 4 The ICU Department of TEDA International Cardiovascular Disease Hospital, No. 61 Third Avenue, Tianjin Economic and Technological Development Zone, Tianjin, China; 5 The Cardiovascular Surgery of TEDA International Cardiovascular Disease Hospital, No. 61 Third Avenue, Tianjin Economic and Technological Development Zone, Tianjin, China

**Keywords:** Acute kidney injury, Coronary artery bypass grafting, Furosemide

## Abstract

**Objectives:**

Furosemide is usually administered before the Coronary artery bypass grafting (CABG) to improve water–sodium retention. However, no final conclusions are available on the postoperative renal outcome of furosemide. We evaluated the effect of preoperative furosemide on acute kidney injury (AKI) after CABG.

**Methods:**

We recorded the use of furosemide 14 days before surgery in all patients who underwent CABG from 2016 to 2017. Patients were divided into furosemide (F) group and non-furosemide (NF) group according to preoperative use of furosemide. A 1:1 propensity score matching was performed. Multivariate analyses were conducted to determine risk factors for AKI after CABG.

**Results:**

Overall, 974 patients were included in the study, of which 82 cases were complicated with postoperative AKI. The incidence of AKI was significantly increased in F group than NF group (28.9% vs. 7.4%, *p* = 0.000). After adjusting for risk factors, the incidence of AKI in the F group was 5.34 times more than the NF group (95% confidence interval [CI] 2.45–11.64; *p* = 0.000). The incidence of AKI increased significantly when the cumulative dosage of furosemide exceeded 110 mg (odds ratio [OR] 6.23; 95% CI 2.07–18.74, *p* = 0.001) and 250 mg (OR 8.31; 95% CI 2.87–24.02, *p* = 0.000). After the propensity-matching group analysis, same results were obtained.

**Conclusions:**

The incidence of AKI after CABG was related to the use of preoperative furosemide, and it increased exponentially with the increase of cumulative dose of furosemide. This provides guidance for the dose of preoperative furosemide.

**Supplementary Information:**

The online version contains supplementary material available at 10.1007/s11748-021-01599-0.

## Introduction

According to data in 2018, the number of cardiovascular disease patients is 290 million in China; cardiovascular death is the leading cause of all-cause death among urban (45.5%) and rural (43.16%) residents [1]. Coronary artery bypass surgery (CABG) is one of the most effective methods to treat coronary heart disease. In 2017, there were 45,455 cases of CABG in China. Acute kidney injury (AKI) is one of the most common and serious complications after CABG [2]. AKI can affect long-term renal function, prolong hospitalization time, and increase economic burden and all-cause mortality [3].The incidence of AKI after cardiac surgery is 5–30%, among which 2–11.2% need renal replacement therapy [4–6].Therefore, it is very important to identify the risk factors of postoperative AKI and take effective preventive measures before CABG.

Furosemide is a commonly used to reduce water–sodium retention during perioperative period [7]. The traditional view is that furosemide can effectively improve urine volume and restore renal function in the early stages of AKI. However, in clinical trail, no benefit of furosemide has been reported on renal function after surgery [8]. Few studies have focus on the relationship between furosemide during surgery of CABG and postoperative renal failure [9, 10]. However, there is little report on the effect of furosemide before CABG on postoperative renal function.

In this retrospective study, we analyze the effect of preoperative furosemide on AKI after CABG. To compare renal injury and outcome between the groups, we chose propensity score matching to balance baseline characteristics.

## Materials and methods

### Patients

Overall, 974 patients with a mean age of 61.9 ± 8.1 years who received primary isolated CABG at TEDA International Cardiovascular Disease Hospital from January 1, 2016 to December 31, 2017 were included in this retrospective study. According to the use of furosemide within 14 days before operation, the patients were divided into furosemide group (F, n = 45) and non-furosemide group (NF, n = 929). We excluded the patients with preoperative use of furosemide over 14 days, dialysis, malignancy, acute infections, emergency operations, repeat CABG, patients who had been administered steroids, patients for whom data such as serum creatinine levels were missing.

The present study was approved by the Medical Ethics Committee of TEDA International Cardiovascular Disease Hospital, which waived the requirement for informed patient consent because of the retrospective nature of the study.

## Surgical technique

In this study, all CABG operations were performed by the same operation and anesthesia team, including 3 surgeons and 2 anaesthesiologists. In this study, aspirin was stopped in all patients 5 days before operation, and statins were taken in all patients. Anesthesia was induced by administration and was maintained by combined intravenous and inhaled anesthesia. The decision of on-pump or off-pump with cardiopulmonary bypass (CPB) was based on the preference of the surgeons. The dosage of heparin was 3 mg/kg if the operation was on-pump and 1 mg/kg if off-pump. Doses of vasoactive drugs were adjusted according to the heart function. Pulmonary static inflation (pressure 5–10 cm H2O) was used during CPB. After the operation, patients were taken to a dedicated cardiovascular intensive care unit (ICU).

## Data collection

The data in this study are from the electronic inpatient medical record management system database of TEDA International Cardiovascular Disease Hospital.

The primary outcome AKI after CABG was defined based on an improved AKI classification method proposed by the Acute Kidney Injury Network (AKIN) classifications, an international cooperative organization of nephrologists and critical physicians [11], which is more sensitive than RIFLE, has no difference in the prediction of in-hospital mortality with KDIGO standard [12], and is widely used in AKI research after CABG [13]. AKIN stage 1: an increase in creatinine to 1.5 times that of baseline or an increase > 0.3 mg/dl within 48 h; stage 2: an increase in the creatinine level to 2 times that at baseline; stage 3: an increase in the creatinine level to 3 times that at baseline. Creatinine levels were routinely measured on the morning of the operation day and at days 1 and 2 after surgery; we used the maximum level within 48 h as the postoperative creatinine level.

In all patients, 2 ml of venous blood was taken from fasting for more than 8 h after admission to determine the blood routine, liver and kidney function, blood glucose, and blood lipid.

CKD-EPI formula [14] was used to calculate preoperative eGFR. Blood pressure of right brachial artery was measured for 3 days before operation, and the mean value was calculated as the preoperative blood pressure. Echocardiography was performed on admission and left ventricular ejection fraction (LVEF) and left ventricular end-diastolic diameter (LVDD) were recorded. Preoperative use of calcium antagonist (CCB), angiotensin receptor antagonists (ARB)/angiotensin-converting enzyme inhibitor (ACEI), β receptor blockers and furosemide were recorded. The cumulative dose of furosemide before operation refers to the product of the daily dose of furosemide and the number of days used within 14 days before operation.

In addition, postoperative hospital stay refers to the time from the first day of operation to discharge. ICU stay refers to the time from the operation to transfer out of ICU. New postoperative atrial fibrillation refers to the detection of atrial fibrillation by postoperative monitoring in patients without atrial fibrillation history. Operative mortality was defined as all deaths that occurred during the hospital stay or after hospital discharge but within 30 days postoperatively. Massive blood transfusion refers to the transfusion of > 5000 mL of blood [15] in 12 h or transfusion of blood that is 1.5 times more than the total blood volume of the patient within 24 h.

## Statistical analysis

A single-sample Kolmogorov–Smirnov test was employed for continuous variables. Normally distributed variables or those that were normally distributed after logarithmic transformation were expressed as mean ± sd and were analyzed using the Student’s *t *test. For variables that were not Skewness distribution, the median (25% and 75%) was presented and non-parametric tests. Categorical data were displayed as frequencies and percentages, and were analyzed using Mann–Whitney rank-sum tests and Chi-square tests.

Initially, univariate logistic regression analysis was performed to identify significant predictors of AKI after CABG. Those variables identified to have a value of p < 0.05 in the univariate analysis, which was performed in a stepwise fashion to identify independent predictors of AKI.

A 1:1 propensity score matching, using New York Heart Association classification (NYHA) and mechanical ventilation time as matching factors, was matched was performed to select patients from the NF group to reduce the effect of preoperative variability between the groups. Matching was performed using greedy, nearest neighbor matching and a caliper of 0.01.

Potential preoperative confounding factors considered in this analysis were selected on the basis of a literature review and clinical plausibility. These variables included (1) demographics characteristics such as age and gender; (2) clinical risk factors such as preoperative eGFR, level of hemoglobin, diabetes mellitus(DM), hypertension, old myocardial infarction(MI), LVEF < 50%, NYHA, preoperative use of an intra-aortic balloon pump (IABP), rate of on-pump CPB and blood transfusion. We included in the analysis preoperative medications such as CCB and ACEI/ARB. Models fit analysis was evaluated with the Hosmer Lemeshow goodness-of-fit statistic. Odds ratios (OR) and their associated 95% confidence intervals (CI) were estimated. In all tests, values of p < 0.05 were considered significant. Statistical analysis was done with SPSS version 25.0 (SPSS Inc., Chicago, IL, USA) was used for the statistical analysis.

## Results

### Patient characteristics

A total of 974 patients were included throughout the study period. Among them, 701 (72.0%) were male. The mean age was 61.9 ± 8.0 years. Baseline characteristics and postoperative outcome data are presented in Table 1. Patients had higher fasting blood glucose, poor NYHA, more OMI, more on-pump, longer mechanical ventilation time; in addition, lower hemoglobin, left ventricular ejection fraction and left ventricular diastolic function in F group. The cumulative dose of furosemide was 189.3 ± 129.5 mg in F group. The cumulative dose of furosemide was closely related to the severity of NYHA before operation, but not to EGFR. Supplement 1. We used NYHA and the time of artificial assisted ventilation as matching variables, and analyzed the 1:1 propensity-matching score between the two groups. In group F, there were no significant differences in baseline characteristics except for lower left ventricular ejection fraction, higher incidence of old myocardial infarction and DM.

**Table 1:** Baseline characteristics of patients

	F group (N = 45)	NF group (N = 929)	*P* value	F group(N = 44)	NF group (N = 44)	*P *value
Age, (y)	63.2 ± 7.5	61.9 ± 8.1	0.290	63.3 ± 7.5	64.7 ± 7.3	0.406
Male, n (%)	33(73.3)	668(71.9)	0.835	33(75.0)	28(73.6)	0.248
BMI, kg/m2	25.9 ± 4.0	26.2 ± 3.2	0.637	26.0 ± 4.0	21.7 ± 2.8	0.000
Systolic pressure, mmHg	123.0 ± 14.1	125.0 ± 13.4	0.332	122.7 ± 14.1	122.5 ± 14.4	0.961
Diastolic pressures, mmHg	70.8 ± 9.6	71.4 ± 8.1	0.636	70.8 ± 9.7	68.1 ± 6.9	0.144
TSH, mIU/L	3.1 ± 5.6	3.0 ± 5.7	0.907	2.1(1.3,2.1)	1.9(1.4,3.3)	0.751
FBG, mmol/l	6.3(5.2,8.3)	5.7(5.0,7.0)	0.033	6.3(5.2,7.4)	5.4(5.0,6.9)	0.092
TC, mmol/l	4.3 ± 1.3	4.5 ± 1.1	0.184	4.3 ± 1.3	4.3 ± 1.3	0.855
TG, mmol/l	1.7 ± 0.9	1.8 ± 1.5	0.400	1.7 ± 1.0	1.6 ± 0.9	0.624
HDL-C, mmol/l	1.0 ± 0.2	1.0 ± 0.2	0.270	1.0 ± 0.2	1.0 ± 0.3	0.388
LDL-C, mmol/l	2.7 ± 1.1	2.8 ± 1.0	0.214	2.7 ± 1.0	2.7 ± 1.1	0.860
Hemoglobin, g/l	132.8 ± 15.5	137.9 ± 15.2	0.026	132.9 ± 15.6	129.2 ± 15.1	0.262
UA, μmol/l	344.4 ± 94.2	329.5 ± 85.1	0.178	348.7 ± 90.7	330.3 ± 82.8	0.293
ALT, u/l	20(15,28)	20(14,32)	0.554	19.5(15,27.8)	20.0(13.3,30.0)	0.963
Baseline eGFR, ml/min/1.73m2	78.2 ± 18.1	81.1 ± 16.1	0.232	77.7 ± 17.8	81.8 ± 15.4	0.372
baseline creatinine, µmol/l	71.4 ± 17.2	69.0 ± 15.3	0.294	71.9 ± 17.1	67.1 ± 13.5	0.151
Peak creatinine postoperation, µmol/l	82(69,99)	71(61,83)	0.008	81.0(69.0,99.5)	66.5(56.0,76.0)	0.000
Smoker, n (%)	32(64.0)	511(55.0)	0.342	28(63.6)	23(52.3)	0.280
Hypertension, n (%)	28(62.2)	647(69.6)	0.171	26(59.1)	30(68.2)	0.114
Diabetes mellitus, n (%)	23(51.1)	326(35.1)	0.029	22(50.0)	16(36.4)	0.197
Hypercholesterolemia, n (%)	10(22.2)	312(33.6)	0.114	10(22.2)	14(31.8)	0.338
NYHA: I	12(26.7)	349(37.6)	0.000	11(23.9)	10(22.7)	0.965
II	20(44.4)	519(55.8)		20(45.5)	21(47.7)	
III–IV	13(28.9)	62(6.7)		13(29.5)	13(29.5)	
Atrial fibrillation, n (%)	5(10.0)	38(4.1)	0.102	5(11.4)	1(2.3)	0.205
Old MI, n (%)	18.0(40.0)	222.0(23.9)	0.014	18.0(40.9)	13.0(29.5)	0.265
LVEF, %	58.0(42.0,61.0)	60.0(57.0,64.0)	0.000	58.0(42.0,61.8)	55.5(47.3,62.0)	0.880
LVDD, mm	51.0(47.0,57.0)	48.0(45.0,51.0)	0.000	51.0(47.0,57.0)	49.0(46.0,55.0)	0.220
EF < 50%, n (%)	19.0(42.2)	65.0(7.0)	0.000	18(40.9)	16(36.4)	0.661
Left main disease n (%)	12.0(26.7)	262.0(28.4)	0.806	11.0(25.0)	11.0(25.0)	1.000
Number of bridging vessels, n (%):						
1	0(0)	26(2.8)	0.073	0(0)	2(4.5)	0.010
2	1 (2.0)	66(7.1)		1(2.3)	4(9.1)	
3	10 (22.2)	278(29.9)		9(20.5)	18(40.9)	
4	34 (75.6)	559(60.2)		34(77.3)	20(45.5)	
**Pre-op medications**						
β-blocker, n (%)	33(73.3)	714(76.9)	0.585	32 (72.7)	32 (72.7)	1.000
CCB, n (%)	7(15.4)	266(28.6)	0.056	7 (15.9)	10 (22.7)	0.418
ARB/ACEI, n (%)	26(57.8)	564(60.7)	0.694	25 (56.8)	24 (54.5)	0830
IABP, n (%)	4(8.9)	28 (3.0)	0.083	4(8.9)	2(4.5)	0.672
On-pump, n (%)	40(88.9)	677 (72.9)	0.017	39(88.6)	32(72.7)	0.059
Massive blood transfusion, n (%)	5(11.1)	57 (6.1)	0.307	5(11.1)	2(4.5)	0.237
mechanical ventilation time, h	15(9,20)	10(6,17)	0.001	15.0(9.3,20.0)	9(7.0,17.5)	0.025
**Postoperative outcomes**						
AKI, n (%)	13(28.9)	69(7.4)	0.000	12(27.3)	3(6.8)	0.011
I	11(24.4)	60(6.5)		10 (22.7)	3(6.8)	0.012
II	1(2.2)	6(0.6)		1 (2.3)	0(0.0)	
III	1(2.2)	3(0.3)		1 (2.3)	0(0.0)	
Postoperative hospital stays, d	8.5(7.4–11.4)	8.4(7.4,10.4)	0.467	8.5(7.4,11.2)	8.4(7.0,10.4)	0.297
ICU stays, h	44(40,69)	43(39,46)	0.135	44.0(39.5,68.5)	43.0(39.3,46.0)	0.282
Death, n (%)	0(0.0)	7 (0.8)	1.000	0(0)	1(2.2)	1.000
New-onset atrial fibrillation, n (%)	2(4.4)	21 (2.3)	0.661	2(4.5)	0(0)	0.093

BMI: body mass index, TSH: thyroid-stimulating hormone, FBG: fasting venous blood sugar, TC: total cholesterol, TG: triglyceride, HDL-C: high-density lipoprotein cholesterol, LDL-C: low-density lipoprotein cholesterol, MI: myocardial infarction, NYHA: New York Heart Association classification method, LVEF: left ventricular ejection fraction, LVEDV: left ventricular end-diastolic volume, eGFR: estimated glomerular filtration rate, UA: uric acid, IABP: intra-aortic balloon pump, CCB: Calcium Antagonists, ARB: Angiotensin II receptor antagonists, ACEI: angiotensin-converting-enzyme inhibitors, AKI: acute kidney injury, ICU: intensive Care Unit

## Renal outcomes

In this study, 82 patients (8.4%) developed AKI after CABG: stage I (*n* = 71, 86.6%), stage II (*n* = 7, 8.5%), and stage III (*n* = 4, 4.9%) patients; no patients needed dialysis. Compared with group NF, the peak creatinine and the incidence of AKI in different grades in group F was significantly increased than that in group NF (*p* = 0.000). After matching, the incidence of AKI in patients who received preoperative furosemide was 34% compared with 8% for patients who did not (*p* = 0.001). However, there was no significant difference in ICU time, hospital stay, new-onset atrial fibrillation and mortality between the two groups before and after matching.

### Independent risk factors for postoperative AKI

The univariate analysis showed that risk factors related with AKI were pre-op eGFR, hypertension, SBP, hemoglobin, the use of IABP, pre-op ACEI/ARB, pre-op furosemide, mechanical ventilation time, on-pump, and massive blood transfusion (Table 2). The stepwise regression analysis of multivariate analysis shows that hypertension, SBP, pre-op furosemide (OR 5.34; 95% CI 2.45–11.64; *p* = 0.000), and mechanical ventilation time were independent risk factors. After adjusting for propensity score and covariates, preoperative furosemide (OR 14.50; 95% CI 2.68–78.6; *p* 0.002) and mechanical ventilation time (OR 1.05; 95% CI 1.02–1.09; *p* = 0.003) was found to increase the incidence of AKI after CABG.

**Table 2:** Logistic regression analysis for risk factors associated with acute kidney injury

Variables	Before matching				After matching			
	Unadjusted	*P*	Adjusted	*P*	Unadjusted	*P*	Adjusted	*P*
	OR (95% CI)	Value	OR (95% CI)	Value	OR (95% CI)	Value	OR (95% CI)	Value
Pre-op eGFR	0.98(0.97–1.00)	0.183			0.99(0.97–1.00)	0.197		
BMI	0.96(0.88,1.04)	0.302			0.94(0.87,1.02)	0.154		
DM	1.26(0.74–2.15)	0.390			1.35(0.78,2.32)	0.283		
Hypertension	1.98(0.94–4.16)	0.071	2.09(1.03–4.22)	0.041	2.10(0.99–4.44)	0.053		
SBP, mmHg	1.03(1.01–1.06)	0.000	1.03(1.01–1.05)	0.001	1.04(1.01–1.06)	0.001		
Old MI	0.99(0.54–1.85)	0.985			1.00(0.54–1.86)	0.999		
Hemoglobin, g/l	0.98(0.97–1.00)	0.033	0.98(0.97,1.00)	0.044	0.99(0.97,1.00)	0.08		
UA, mmol/l	1.00(1.00–1.01)	0.006	1.00(1.00–1.01)	0.001	1.00(0.99–1.00)	0.003		
NYHA(I–IV)	0.91(0.58–1.41)	0.658			0.93(0.60–1.45)	0.751		
LVEF < 50%	0.50(0.18–1.41)	0.504			0.45(0.16–1.30)	0.141		
IABP	2.94(0.98–8.84)	0.055			2.89(0.95–8.74)	0.061		
Pre-op ACEI/ARB	1.207(0.66–2.21)	0.541			1.21(0.66–2.22)	0.532		
Pre-op furosemide	6.20(2.65–14.53)	0.000	5.21(2.39–11.35)	0.000	6.43(2.72–15.17)	0.013	6.47(1.60–26.27)	0.009
On pump	1.58(0.78–3.21)	0.208			1.59(0.78–3.24)	0.199		
Mechanical ventilation time, h	1.03(1.02–1.02)	0.000	1.03(1.02–1.04)	0.000	1.03(1.02–1.04)	0.000	1.05(1.02–1.09)	0.001
Massive blood transfusion	1.35(0.56–3.28)	0.507			1.28(0.53–3.13)	0.587		

*eGFR: estimated glomerular filtration rate, UA: uric acid, DM: diabetes mellitus, SBP: systolic blood pressure, MI: myocardial infarction, NYHA: New York Heart Association classification method, LVEF: left ventricular ejection fraction, IABP: intra-aortic balloon pump, ARB: Angiotensin II receptor antagonists, ACEI: angiotensin-converting enzyme inhibitors, AKI: Acute Kidney injury

The multivariate model significantly predicted the occurrence of AKI (model *χ*^2^, 125.138; *p* = 0.000). The model was well calibrated among deciles of observed and expected risk (Hosmer–Lemeshow *χ*^2^ = 11.591; *p* = 0.170).

### The effect of cumulative dose of furosemide on AKI

All patients in F group were divided into three groups according to the cumulative dose of furosemide 14 days before operation: < 110 mg (*n* = 15), ≥ 110—< 250 mg, *n* = 15; and ≥ 250 mg, *n* = 15. With the increasing doses of furosemide, AKI prevalence increased gradually (*χ*^2^ = 33.135, *p* = 0.000). There was no difference in AKI between patients with cumulative dose less than 110 mg and NF group. When the cumulative dose exceeds 110 mg, the incidence of AKI will increase significantly (OR 6.23; 95% CI 2.07–18.74, *p* = 0.001). If the cumulative dose reaches 250 mg, the risk is 8 times higher than those without furosemide (OR 8.31; 95% CI 2.87–34.02, *p* = 0.000). Similar conclusions are drawn from the analysis of matched data. When the cumulative dose exceeds 110 mg, the incidence of AKI will increase significantly (OR 4.00; 95% CI 0.97–18.74, *p* = 0.056). If the cumulative dose reaches 250 mg, the risk is times higher than those without furosemide (OR 5.33; 95% CI 1.33–21.41, *p* = 0.018) (Table 3).

**Table 3:** The effect of different doses of furosemide before CABG on the occurrence of AKI

	Before matching				After matching			
	No-AKI	AKI	OR (95% CI)	*P* value	No-AKI	AKI	OR (95% CI)	*P* value
0 mg (n, %)	860(92.6)	69(7.4)		0.000	40(88.9)	5(11.1)		0.045
< 110 mg (n, %)	13(86.7)	2(13.3)	1.92(0.424, 8.67)	0.398	13(86.7)	2(13.3)	1.23(0.21–7.12)	0.817
≥ 110- < 250 mg (n, %)	10(66.7)	5(33.3)	6.23(2.07, 18.74)	0.001	10(66.7)	5(33.3)	4.00(0.97–16.55)	0.056
≥ 250 mg (n, %)	9(60)	6(40.0)	8.31(2.87, 24.02)	0.000	9(60.0)	6(40.0)	5.33(1.33–21.41)	0.018

*CABG* coronary artery bypass grafting, *AKI* acute renal injury

## Discussion

According to different diagnostic criteria, AKI related to cardiac surgery is a common postoperative complication: the incidence is 4–9%, the related mortality rate was 12.6% [16–18]. The cause for the increase in mortality during hospitalization due to AKI after CABG is unknown, which may be related to the overload of capacity, metabolic acidosis, electrolyte imbalance, and increased risk of infection [19, 20]. In this study, the prevalence of AKI after CABG was 8.4%, and the mortality rate was 8.5% in patients with AKI; no deaths in patients without AKI were reported. Most of them were classified as stage I (*n* = 71, 86.6%). There were no patients on dialysis. The incidence of AKI in this study was lower than that in previous studies. It may be related to the different diagnostic criteria and the stricter control of surgical indications.

Furosemide inhibits the reabsorption of water and electrolytes by the renal tubules and thus increases urine output. In recent clinical studies, the effect of furosemide on postoperative renal function is controversial. Solmaz Fakhari found that intra- and early postoperative furosemide infusion has a renal protective effect in adult cardiac surgery with cardiopulmonary bypass [21]. However, other studies suggest that whether single dose or continuous infusion of furosemide was added to patients’ blood at the beginning of CBP without any protective effect on postoperative renal function [22]. A multicenter study of 552 patients with AKI showed that the use of diuretics significantly increased the rate of in-hospital mortality [4]. However, no conclusions are available on the effect of preoperative furosemide therapy on renal outcome in CABG patients. The definition of furosemide’s impact on postoperative renal dysfunction should add important benefits to CABG outcomes.

This analysis found that preoperative furosemide are associated with a significantly high risk of postoperative AKI compared with no preoperative furosemide treatment (28.9% vs. 7.4%). After matching, the incidence of AKI in group F was still higher than that in group NF (27.3% vs. 6.8%). After adjusting for DM, BMI, NYHA, OMI, eGFR, ARB, blood transfusion and on-pump, hypertension history, elevated systolic blood pressure, decreased hemoglobin, furosemide and prolonged mechanical ventilation time were risk factors for AKI after CABG. Moreover, furosemide and prolonged mechanical ventilation time were still risk factors after matching. We observed a over fivefold increase in AKI risk. Most previous studies focus on a deleterious effect of furosemide applicated during operation on renal function after operation [11, 23]. Few studies have reported on the effect of preoperative furosemide. Loubon et al. [25] reported that the proportion of patients taking diuretics for a long time after AKI was significantly higher than that of patients without AKI (37% vs. 19.9%, *p* = 0.005).Their results support our conclusion. However, there is no evidence that diuretic use is a risk factor for AKI in their study. This study confirmed for the first time that the application of furosemide before operation is related to the occurrence of AKI after operation.

According to the group analysis of different cumulative doses of furosemide before surgery, the incidence of AKI increased with the increase in the cumulative dose of furosemide. Lombardi and his colleagues [25] also found that furosemide-related decreased renal function after cardiac surgery was related to its dose. When the cumulative dose of furosemide was more than 110 mg, AKI increased over 6 times; when the cumulative dose was more than 250 mg, AKI nearly increased 8 times. This result needs to be verified by expanding the sample, which will provide important guidance for clinical treatment.

Other independent risk factors for AKI identified in the present analysis were consistent with other reports. We found postoperative AKI was strongly related with hypertension [4], preoperative mean SBP, and mechanical ventilation time [28] were all independent risk factors for AKI. In this study, although there were differences in the duration of mechanical ventilation, hemoglobin and cardiac function classification between the two groups at baseline, furosemide and prolonged mechanical ventilation time were still independent risk factors for AKI after CABG after propensity-matching eliminated these differences. In this study, we failed to find that perioperative hemodynamic instability including the reduction of LVEF [29] and cardiac function grading were independent determinants of AKI. These differences might be due to the different definitions of left ventricular ejection fraction decline (< 50% vs. < 30%).

## Limitations

This study had some limitations. First, this is a single-center retrospective study. A similar study needs to be conducted in a multicenter prospective study to prove its wide applicability. Although this study is a central study, patients from different provinces and cities in China are still represented. Second, our model is only for isolated CABG, and the impact of more complex combined heart surgery still needs further study. Lastly, the retrospective nature of this study and the small number of patients limited the validity of the clinical outcome. We only observed the effect of cumulative dose of furosemide 14 days before operation on postoperative AKI, so we need to continue to follow-up to observe the long-term changes of renal function. Nevertheless, although larger samples would be needed to produce more accurate and convincing results, this study already presents interesting findings related to postoperative outcomes.

## Conclusion

The present propensity score-adjusted analysis showed that preoperative furosemide increases the risk of AKI after CABG. Moreover, this effect increased with the increase in the preoperative cumulative use of furosemide. These results suggest that the dose of furosemide should be strictly controlled before CABG. However, an appropriately powered, randomized, controlled trial evaluating the optimal management of preoperative furosemide therapy before CABG operations would be useful to confirm our results.

## Supplementary Information

Below is the link to the electronic supplementary material.

Supplementary file1 (DOCX 19 KB)

## Abbreviations

AKI: Acute kidney injury
AKIN: Acute kidney injury network
CABG: Coronary artery bypass grafting
CPB: Cardiopulmonary bypass
eGFR: Estimated glomerular filtration rate
IABP: Intra-aortic balloon pumping
SD: Standard deviation
BMI: Body mass index
TSH: Thyroid-stimulating hormone
FBG: Fasting venous blood sugar
TC: Total cholesterol
TG: Triglyceride
HDL-C: High-density lipoprotein cholesterol
LDL-C: Low-density lipoprotein cholesterol
MI: Myocardial infarction
NYHA: New York Heart Association classification method
LVEF: Left ventricular ejection fraction
LVEDV: Left ventricular end-diastolic volume
UA: Uric acid
CCB: Calcium antagonists
ARB: Angiotensin II receptor antagonists
ACEI: Angiotensin-converting-enzyme inhibitors
ICU: Intensive care unit
