# Supplementary material for: Preoperative use of furosemide may increase the incidence of acute kidney injury after coronary artery bypass grafting: a propensity score-matched study
Source: Gen Thorac Cardiovasc Surg. 2021 Feb 6;69(10):1392–9. doi: 10.1007/s11748-021-01599-0 (PMC8417004; doi:10.1007/s11748-021-01599-0)
Supplement: Supplementary file 1 — Supplementary file1 (DOCX 19 KB) [file 11748_2021_1599_MOESM1_ESM.docx]

Supplement 1

45 patients who used preoperative furosemide were divided into three levels: <110 mg，n = 15; ≥110 - <250 mg, n = 15; and ≥250 mg, n = 15. (Table 4) The reason for using furosemide during hospitalization was cardiac insufficiency. The difference of cumulative dose of furosemide between the patients of NYHA grade I, II and III-IV was statistically significant. With the increase of NYHA level, the proportion of cumulative dose of high dose furosemide is also increased. However，there was no significant difference in the cumulative dose of furosemide between the eGFR ≥ 60 ml/min/1.73m^2^ group and eGFR < 60 ml/min/1.73m^2^ group.（Table 5）

Table 4 Comparison of cumulative dose of furosemide in patients with different NYHA levels

|  | Before matching | | |  | After matching | | |  |
| --- | --- | --- | --- | --- | --- | --- | --- | --- |
|  | NYHA:I | II | III-IV | *P* Value | NYHA:I | II | III-IV | *P* Value |
| 0mg(n,%) | 349(37.6) | 518(55.8) | 62(6.7) | 0.000 | 12(26.7) | 20(44.4) | 13(28.9) | 0.070 |
| <110mg(n,%) | 6(40) | 6(40.0) | 3(20.0) |  | 6(40.0) | 6(40.0) | 3(20.0) |  |
| ≥110-<250mg(n,%) | 2(13.3) | 11(73.3) | 2(13.3) |  | 2(13.3) | 11(73.3) | 2(13.3) |  |
| ≥250mg(n,%) | 4(26.7) | 3(20.0) | 8(53.3) |  | 4(26.7) | 3(20.0) | 8(53.3) |  |

According to the pre-operative eGFR, the patients were divided into two groups: eGFR ≥ 60 ml/min/1.73m^2^ group and eGFR < 60 ml/min/1.73m^2^ group. There was no significant difference in the cumulative dose of furosemide between the two groups.

Table 5 Comparison of cumulative dose of furosemide in patients with different eGFR level

|  | eGFR ≥ 60 ml/min/1.73m^2^ | eGFR < 60 ml/min/1.73m^2^ | P value |
| --- | --- | --- | --- |
| 0mg | 829(89.2) | 100(10.8) | 0.098 |
| <110mg | 11(73.3) | 4(26.7) |  |
| ≥110-<250mg | 14(80) | 4(20) |  |
| ≥250mg | 13(86.7) | 2(13.3) |  |
